# Toxic Shock Syndrome Toxin-1 (TSST-1) in *Staphylococcus aureus*: Prevalence, Molecular Mechanisms, and Public Health Implications

**DOI:** 10.3390/toxins17070323

**Published:** 2025-06-24

**Authors:** Rahima Touaitia, Nasir Adam Ibrahim, Eman Abdullah Almuqri, Nosiba S. Basher, Takfarinas Idres, Abdelaziz Touati

**Affiliations:** 1 Department of Natural and Life Sciences, Faculty of Exact Sciences and Natural and Life Sciences, University of Tebessa, Tebessa 12000, Algeria; rahima.touaitia@univ-tebessa.dz; 2 Department of Biology, College of Science, Imam Mohammad Ibn Saud Islamic University (IMSIU), Riyadh 13318, Saudi Arabia; naabdalneim@imamu.edu.sa (N.A.I.); eaalmuqri@imamu.edu.sa (E.A.A.); nsbasher@imamu.edu.sa (N.S.B.); 3 Laboratory for Livestock Animal Production and Health Research, Rabie Bouchama National Veterinary School of Algiers, Issad ABBAS Street, BP 161 Oued Smar, Algiers 16059, Algeria; 4 Laboratoire d’Ecologie Microbienne, Faculté des Sciences de la Nature et de la Vie (FSNV), Université de Bejaia, Bejaia 06000, Algeria; abdelaziz.touati@univ-bejaia.dz

**Keywords:** *Staphylococcus aureus*, TSST-1, toxic shock syndrome, detection methods, antimicrobial resistance, public health

## Abstract

*Staphylococcus aureus* is a significant pathogen responsible for various infections, with its production of toxic shock syndrome toxin-1 (TSST-1) being a central factor in the pathogenesis of toxic shock syndrome (TSS). This study investigates the prevalence, molecular mechanisms, and public health implications of TSST-1-producing *S. aureus*. This study reviews methods for detecting TSST-1, focusing on PCR-based molecular techniques and immunological methods like ELISA, as well as the challenges in accurately diagnosing TSST-1 due to antibiotic resistance and strain variability. The findings reveal that TSST-1 is widely distributed across clinical, foodborne, and zoonotic sources, with significant prevalence in both healthcare and agricultural settings. This study also discusses the regulatory networks controlling TSST-1 production, including the *agr* system and other environmental cues like glucose, iron, and pH levels, which influence toxin expression. The results underline the need for improved surveillance and diagnostic approaches, as well as the development of targeted therapies to mitigate the impact of TSST-1 in both hospital and community settings. The conclusions highlight the importance of understanding TSST-1’s molecular mechanisms for developing effective public health strategies to control its spread.

## 1. Introduction

*Staphylococcus aureus* is a versatile Gram-positive pathogen responsible for a broad spectrum of infections, ranging from localized skin conditions to life-threatening systemic diseases such as sepsis, pneumonia, and toxic shock syndrome (TSS) [1,2]. Central to its virulence is the production of superantigens (SAgs), a class of exotoxins that subvert host immune responses by inducing non-specific T-cell activation. Among these, toxic shock syndrome toxin-1 (TSST-1) is a pivotal virulence factor historically linked to menstrual TSS (mTSS) outbreaks in the 1980s, particularly associated with high-absorbency tampon use [3,4,5]. TSST-1 remains a critical focus in clinical and microbiological research due to its role in both menstrual and non-menstrual TSS, including cases arising from surgical wounds, burns, and other trauma sites [6,7].

TSST-1 exerts its effects by binding to major histocompatibility complex class II (MHC-II) molecules on antigen-presenting cells and specific Vβ regions of T-cell receptors (TCR), bypassing conventional antigen processing. This interaction triggers polyclonal T-cell activation, leading to a cytokine storm characterized by excessive release of pro-inflammatory cytokines such as TNF-α, IL-1, and IFN-γ. The resultant systemic inflammation drives the hallmark symptoms of TSS: fever, hypotension, rash, and multi-organ failure [8,9,10]. TSST-1’s superantigenic activity is regulated by the accessory gene regulator (Agr) system and other transcriptional networks, which modulate toxin expression in response to environmental cues, such as oxygen levels and nutrient availability [11,12].

TSST-1-producing *S. aureus* strains are implicated in severe clinical outcomes, particularly in methicillin-resistant *S. aureus* (MRSA) infections. These strains are prevalent in clonal complexes such as CC5 and ST22, contributing to high mortality rates in bacteremia and sepsis [13,14]. Beyond TSS, TSST-1 exacerbates conditions like eczema herpeticum, necrotizing pneumonia, and septic arthritis, underscoring its systemic pathogenic potential [15,16]. Epidemiologically, TSST-1-producing strains are globally distributed, with reported cases in clinical, foodborne, and zoonotic contexts. For instance, TSST-1 has been detected in MRSA isolates from livestock, raw seafood, and dairy products, highlighting its public health risks [17,18,19].

The detection of TSST-1 relies on molecular assays targeting the *tst* gene or immunoassays for toxin identification. However, its association with antibiotic resistance complicates treatment, particularly in MRSA infections [20,21]. Current therapeutic strategies focus on neutralizing TSST-1’s effects, including monoclonal antibodies (e.g., MS473 scFv) and vaccines (e.g., recombinant TSST-1 variant), which have shown promise in preclinical models [22,23]. Preventive measures, such as improved tampon safety standards and food hygiene protocols, remain critical in reducing TSST-1-associated morbidity [24,25].

TSST-1 exemplifies the interplay between bacterial virulence and host immune dysregulation. Its historical association with TSS outbreaks and ongoing relevance in antimicrobial resistance and food safety underscore the need for continued research into its molecular mechanisms, epidemiology, and targeted interventions [3,26].

## 2. Prevalence of TSST-1-Producing *S. aureus*

The global distribution of *S. aureus* strains producing TSST-1 reveals striking geographical variation shaped by regional differences in microbial ecology, healthcare practices, animal husbandry, food production systems, and antimicrobial usage. The following table (Table 1) consolidates prevalence rates reported in diverse populations and sample types across multiple countries.

### 2.1. Africa

The prevalence of TSST-1 producing *S. aureus* across Africa exhibits regional variability, with significant findings reported in clinical, zoonotic, and environmental contexts. In Algeria, TSST-1 prevalence ranges from 16.7% to 22.2%, detected in diverse sources such as human nasal swabs, livestock, pets, and hospital environments, underscoring its zoonotic potential and widespread environmental circulation [27,28,57]. Similarly, Egypt reports TSST-1 in clinical isolates from bacteremic and infective endocarditis patients (21%), wound and sputum samples (unquantified isolates), and multidrug-resistant MRSA from retail oysters, highlighting its presence in both healthcare and foodborne transmission routes [19,30,31].

Notably, TSST-1 is prevalent in food-related reservoirs. In Burkina Faso, MRSA isolates from ready-to-eat food products harbored the toxin, linking food safety to public health risks [58,59]. Ethiopia demonstrates alarming contamination in dairy products, with 51% of isolates from raw milk testing positive, emphasizing risks in food production chains [40]. Clinical settings also show concerning trends: Ethiopia reported 13.37% TSST-1 positivity in wound, blood, and ear swabs [39], while Kenya identified 23.3% prevalence in hospital isolates, particularly in surgical wards, suggesting nosocomial transmission risks [46].

Lower prevalence rates are documented in Benin (7.14%) in pediatric and maternity hospital strains [29], Ghana (11.4%) in cefoxitin-resistant isolates from children with sickle cell disease [60], and Rwanda (low prevalence in bovine mastitis samples) [61]. These variations may reflect differences in sampling methodologies, population demographics, or regional antimicrobial use patterns.

### 2.2. Asia

The prevalence of TSST-1-producing *S. aureus* across Asia varies significantly by country, strain type, and clinical context, reflecting diverse epidemiological patterns. This synthesis consolidates findings from studies conducted in multiple Asian regions, highlighting key trends and variations [62].

China demonstrates considerable heterogeneity in TSST-1 prevalence. Among MRSA isolates, rates range from 2.80% in community and animal samples to 18.0% in hospital-associated CC5 MRSA strains [36,63]. Notably, ST22 MRSA strains form a distinct clade harboring TSST-1 that is absent in European counterparts, underscoring regional genetic divergence [64]. Lower prevalence is observed in bovine mastitis (3.2%) and community-acquired MRSA (CA-MRSA) from pneumonia patients (3.8%), suggesting niche-specific dissemination [34,35].

Iran exhibits a wide spectrum of TSST-1 occurrence, with the highest rate (27.59%) in mastitis milk samples, linked to *mecA*-positive nasal carriers [65]. Clinical settings also show variability: 12.9% in burn patient isolates, 10.97% in hemodialysis patients, and 3.9% in seafood samples [39,66,67]. Interestingly, TSST-1 prevalence in atopic dermatitis (AD) patients declined to 9.7%, with no significant difference between multiple sclerosis patients and controls [42,68].

Japan reports a temporal decline in TSST-1-positive MRSA, from 33.6% (2010) to 21.6% (2018) in outpatient isolates, potentially linked to improved infection control [45]. TSST-1, often co-occurring with Panton-Valentine leukocidin (PVL) genes, is associated with severe skin infections and neonatal intensive care unit (ICU) outbreaks [44,69].

Other Asian regions show distinct patterns. Afghanistan has an exceptionally high prevalence (68.4%) of MRSA isolates from university students, dominated by CC22-MRSA-IV strains [7]. India identifies novel ST22 MRSA sublineages with TSST-1, posing risks for severe infections despite unreported prevalence [70]. South Korea reports TSST-1 in 13.3% of bovine MRSA isolates, marking its first detection in dairy farms [53]. Myanmar and Nepal highlight zoonotic and familial transmission risks, with TSST-1 detected in food handlers (3.5%) and CA-MRSA causing necrotizing pneumonia, respectively [4,71].

### 2.3. Europe

Recent studies have shown that the prevalence of TSST-1-producing *S. aureus* across Europe varies significantly depending on the bacterial strain, host reservoirs, and clinical contexts. In Russia, TSST-1 was detected in 1.6% of *S. aureus* isolates from cows with subclinical mastitis, highlighting its role in veterinary infections [33]. Strikingly, the same country reported a markedly higher prevalence (58%) among MRSA ST239Kras strains, underscoring the toxin’s association with antibiotic-resistant lineages [52].

Poland demonstrated diverse reservoirs for TSST-1 producers. While 3.5% of poultry isolates from broiler chickens and turkeys tested positive [50], wildlife studies identified TSST-1 in 5% of foxes and martens, suggesting rare zoonotic reservoirs [49]. Human health data from Poland revealed an 8% prevalence in atopic dermatitis (AD) patient isolates, with higher rates in adults compared to children, implicating TSST-1 in chronic dermatological conditions [51].

Zoonotic potential was further emphasized in Italy, where 5.9% of *S. aureus* isolates from small ruminants in Sicily carried TSST-1 [43]. Similarly, Portugal reported a 4.8% prevalence in raw milk isolates, linking enterotoxigenic *S. aureus* to food safety risks [9].

In the UK, epidemiological studies highlighted TSST-1’s clinical impact. Although prevalence data were not explicitly reported, TSST-1-associated TSS cases were linked to non-menstrual TSS (nmTSS), particularly in individuals with compromised skin barriers, accounting for 41% of the nmTSS cases [55,72].

### 2.4. Americas

The prevalence of TSST-1-producing *S. aureus* in the Americas exhibits regional and contextual variability influenced by environmental, clinical, and production-related factors.

In Brazil, TSST-1 prevalence in dairy products varies significantly by product type and production practices. A high prevalence of 52.08% was reported in *Minas Frescal* cheese, highlighting contamination risks in dairy processing [32]. Conversely, studies on artisanal *coalho* cheese and raw milk cheeses detected no TSST-1 in food isolates, despite diverse virulence genes [18,30]. This discrepancy suggests that artisanal methods or regional microbial ecology may mitigate toxin production. However, TSST-1 was identified in 2.6% of cheese isolates linked to raw milk handlers, emphasizing contamination risks during artisanal production [31].

In the U.S., TSST-1 research focuses on clinical, genetic, and zoonotic contexts. TSST-1 was detected in 17 animal isolate genomes, including cats, dogs, and cows, underscoring its zoonotic potential [73]. Clinically, the toxin’s role in menstrual TSS is notable: 100% of USA200 lineage strains associated with menstrual TSS cases carry TSST-1, indicating lineage-specific toxin exclusivity [74]. Additionally, 5% of vaginal colonizers among tampon users harbored TSST-1-producing *S. aureus* [75]. Mechanistic studies revealed TSST-1’s regulatory dynamics in *S. aureus* MN8 and its impact on vaginal epithelial cells, influencing host inflammatory responses [76,77].

### 2.5. Epidemiology of TSST-1-Producing S. aureus

The epidemiological data on TSST-1-mediated TSS reveal distinct geographic and demographic patterns. In the United States, the incidence of menstrual TSS (mTSS) linked to TSST-1-producing S. aureus has declined significantly since the 1980s following regulatory changes concerning tampon absorbency and public awareness campaigns, stabilizing at 0.3–0.5 cases per 100,000 menstruating individuals [6,78]. Non-menstrual TSS (nmTSS), however, now represents nearly half of all reported cases and is often associated with surgical infections, postpartum complications, or skin trauma [79]. Mortality rates for staphylococcal TSS remain low overall (3–5%), but nmTSS shows a notably higher mortality rate of up to 22%, likely due to delayed diagnosis and comorbidities [80]. In the United Kingdom, national surveillance reports indicate an overall TSS incidence rate of 0.07 per 100,000, predominantly driven by TSST-1-producing clonal complex 30 (CC30) methicillin-sensitive S. aureus strains, with mortality patterns similar to those observed in the United States [72]. Across a study, morbidity following TSS was consistently associated with organ-specific complications, notably renal, cardiovascular, hepatic, and neurological dysfunctions. A significantly increased rehospitalization rate in men with TSS, accompanied by elevated risks for renal and autoimmune diseases, was observed [81].

Demographic stratification highlights disparities in susceptibility and clinical outcomes. mTSS disproportionately affects adolescents and young adults aged 13–24 years, particularly those using high-absorbency tampons or menstrual cups [82,83,84]. A North American study involving 3012 menstruating women found that Black women colonized with TSST-1-producing *S. aureus* had significantly lower neutralizing anti-TSST-1 antibody titers compared to White and Hispanic women (89% vs. 98% and 100%, respectively), suggesting increased vulnerability [62]. This ethnic variation may be partly explained by differences in the host immune response, including variations in HLA class II alleles that regulate antibody formation [85]. In pediatric populations, TSS generally exhibits a lower mortality rate than in adults, although nmTSS cases are more frequently linked to burns or skin infections in children [84,86].

Emerging research has also focused on preventative measures. A recombinant TSST-1 vaccine (rTSST-1v) has shown promise in recent phase II clinical trials, inducing protective immunity in over 80% of participants after a single dose and maintaining robust antibody levels over time. If validated in phase III trials, this vaccine could offer long-term protection, particularly for at-risk groups such as menstruating adolescents [87].

Table 2 provides a comparative synthesis of findings from multiple recent studies investigating the morbidity and mortality outcomes associated with TSS.

## 3. Methods of Detection

### 3.1. Molecular Methods

#### 3.1.1. PCR-Based Detection

PCR is the cornerstone of TSST-1 detection, leveraging primers specific to the *tst-1* gene to amplify conserved regions. DNA extraction methods vary: studies often use rapid boiling protocols (e.g., 95 °C thermal lysis) for clinical isolates [48,53], while others employ commercial kits or phenol–chloroform purification for higher yields [40,47]. Conventional PCR typically amplifies *tst-1* fragments of 271–350 bp using primers validated for specificity [50,91]. For instance, a 271 bp product confirmed TSST-1 in mastitis milk isolates [65], while a 350 bp amplicon identified *tst-1* in MRSA [92].

Multiplex PCR enhances efficiency by co-amplifying *tst-1* with other virulence (*sea* and *hlb*) or resistance genes (*mecA*), enabling comprehensive profiling. One study screened 23 virulence genes alongside *tst-1* in MRSA isolates [53], while another combined *tst-1* with enterotoxins and *sak* in a single reaction [50]. This approach is particularly valuable in clinical diagnostics, where the rapid identification of toxin profiles guides treatment [2].

Quantitative methods, such as real-time PCR (qPCR), offer both sensitivity and quantification. For example, SYBR Green-based qRT-PCR was used to measure *tst-1* expression in milk-derived *S. aureus* [93], while probe-based assays (e.g., TaqMan) improved specificity in nasal MRSA isolates [48]. Despite these advantages, PCR remains genotypic; studies highlighted the absence of protein validation (e.g., ELISA) as a limitation, risking false positives from silent genes [53,57].

#### 3.1.2. Sequencing and Genomic Approaches

Whole-genome sequencing (WGS) provides unparalleled resolution for *tst-1* characterization. By analyzing entire genomes, WGS identifies *tst-1* within pathogenicity islands, phage integration sites, or plasmids, elucidating its horizontal transfer. For example, WGS linked *tst-1* to SCCmec V in Chinese ST22-MRSA strains, distinguishing them from SCCmec IV-bearing EMRSA-15 lacking the toxin [64]. Another study revealed duplicated *tst-1* genes in ST22-PT clones, suggesting evolutionary adaptations enhancing virulence [94]. WGS also quantifies prevalence, as seen in neonatal bacteremia isolates overexpressing *tst-1* compared to reference genomes [95].

DNA microarrays complement WGS for high-throughput screening. The Alere StaphyType array, which tests 334 markers, detected *tst-1* in 66 *S. aureus* isolates with diverse resistance profiles [57]. However, microarrays are limited to predefined targets, potentially missing novel variants, and lack protein-level confirmation.

Amplicon sequencing resolves ambiguities in PCR results. Sanger sequencing of tst-1 products confirmed gene identity in MRSA [36], while next-generation sequencing (NGS) of multiplex PCR amplicons enabled large-scale surveillance [3]. These methods are critical for distinguishing tst-1 from homologous genes and validating phylogenetic relationships, as demonstrated in global strain comparisons [96].

### 3.2. Immunological Methods

#### 3.2.1. Enzyme-Linked Immunosorbent Assay (ELISA)

ELISA is a cornerstone method for detecting and quantifying TSST-1 in *S. aureus*. This technique relies on the specific binding of antibodies to the toxin [85]. In a sandwich ELISA format, captured antibodies, such as rabbit anti-TSST-1, are immobilized on a plate to bind TSST-1 from samples like bacterial supernatants or serum. Detection antibodies, such as chicken anti-TSST-1 immunoglobulin Y conjugated to enzymes like horseradish peroxidase, are applied, producing a measurable colorimetric signal proportional to the toxin concentration [87,97]. ELISA has been instrumental in quantifying TSST-1 levels in *S. aureus* strains grown under sub-inhibitory concentrations of clindamycin, revealing dose-dependent suppression of toxin production [98]. Additionally, it plays a critical role in evaluating vaccine efficacy, such as measuring TSST-1-specific antibody titers in participants immunized with the rTSST-1v vaccine, thereby assessing immune response durability [87]. Its high specificity and adaptability to high-throughput screening make it indispensable for research and clinical diagnostics, and it is often used alongside Western blotting to confirm toxin presence in clinical samples like blister fluids [14].

#### 3.2.2. Passive Latex Agglutination

Passive latex agglutination is a rapid, cost-effective method for initial TSST-1 screening. This technique employs latex beads coated with anti-TSST-1 antibodies, which agglutinate visibly when exposed to toxin-containing samples, such as bacterial culture supernatants. With a detection limit of 1–2 ng/mL, it is particularly useful for quick assessments [97]. While less sensitive than ELISA, it is frequently paired with the latter for complementary workflows. Latex agglutination provides rapid qualitative results, while ELISA offers precise quantification. This combination enhances diagnostic efficiency, especially in resource-limited settings.

#### 3.2.3. Western Blot and Immunoblot Analysis

Western blot and immunoblot analysis are critical for confirming TSST-1 presence and evaluating its expression. Proteins from bacterial lysates or supernatants are separated via sodium dodecyl sulfate–polyacrylamide gel electrophoresis (SDS-PAGE), transferred to a membrane, and probed with anti-TSST-1 antibodies. Detection methods, such as chemiluminescence, validate the toxin’s presence and molecular weight. For example, immunoblot analysis demonstrated TSST-1 production in 92% of bullous pemphigoid lesion isolates compared to 33% of controls, underscoring its clinical relevance [99]. This method also quantified TSST-1 in blister fluids at concentrations exceeding thresholds required for superantigen activity. Though labor-intensive, its high specificity and ability to detect post-translational modifications make it a valuable confirmatory tool that is often integrated with PCR (*tst* gene detection) and ELISA for comprehensive diagnostics [100].

#### 3.2.4. Functional Assays for Neutralizing Antibodies and Cytokine Profiling

Functional assays for neutralizing antibodies and cytokine profiling extend beyond toxin detection to evaluate immune responses. Neutralizing antibodies, such as those induced by the rTSST-1v vaccine, are assessed for their ability to inhibit TSST-1-mediated T-cell activation. This is measured through T-cell proliferation assays and reductions in pro-inflammatory cytokines like IL-2, IL-6, and TNF-α, providing insights into binding and functional antibody efficacy [85,87]. Cytokine profiling, often conducted via in vitro splenocyte stimulation models, measures cytokines such as IFN-γ, TNF-α, and IL-6 to study immune modulation. For instance, pre-treatment with tofacitinib revealed dose-dependent suppression of TSST-1-driven cytokine release, highlighting its potential to mitigate hyperinflammatory responses [101]. These assays bridge pathogenicity with immune outcomes, offering critical insights for vaccine development and therapeutic interventions.

### 3.3. Other Methods

#### 3.3.1. Phenotypic Assays

Phenotypic assays rely on biochemical and culture-based techniques to identify *S. aureus* and confirm TSST-1 production. Conventional methods include blood agar and MacConkey agar plating for bacterial isolation, followed by coagulase testing to differentiate coagulase-positive (CPS) and coagulase-negative staphylococci (CoNS) [102]. Toxin detection is achieved through reversed passive latex agglutination (RPLA), which uses antibody-coated latex particles to agglutinate in the presence of TSST-1 or enterotoxins. While this method confirmed TSST-1 in MRSA isolates from vaginal discharge [103], it failed to detect toxins in some clinical samples despite TSS symptoms, highlighting variability in toxin expression [104]. Latex agglutination tests on vaginal swab cultures further validated TSST-1 in MSSA [105]. These assays are cost-effective but may lack sensitivity in low-toxin scenarios.

#### 3.3.2. Advanced Proteomic Methods

Advanced proteomics, such as liquid chromatography-selected reaction monitoring (LC-SRM), enables the precise detection and quantification of TSST-1 in complex biological matrices like menstrual fluid [106]. This method identifies signature tryptic peptides unique to TSST-1 and other enterotoxins (SEA, SEC, and SED) using stable isotope-labeled toxins as internal standards. Pre-analytical protocols, including optimized protein digestion and LC-SRM analysis, enhance sensitivity for low-abundance toxins [107]. This approach surpasses traditional immunoassays by providing multiplexed toxin profiling with high specificity, which is critical for studying toxin dynamics in host environments.

#### 3.3.3. Bead-Based Flow Cytometry Assay

A high-sensitivity bead-based flow cytometry assay employs engineered biotinylated Vβ domains immobilized on streptavidin-coated fluorescent beads to selectively capture TSST-1. Detection involves polyclonal anti-TSST-1 antibodies and Alexa Fluor 647-labeled secondary antibodies, achieving a detection limit of 25 pg/mL—10-fold more sensitive than ELISA (0.1–0.25 ng/mL) [108]. The assay supports multiplexing with other toxin-specific Vβ domains, maintaining specificity despite minor cross-reactivity. This technology is ideal for clinical and research settings requiring rapid, high-throughput toxin screening [109,110].

#### 3.3.4. Functional and Imaging-Based Analyses

Functional studies combine imaging and cell-based assays to explore TSST-1 mechanisms. Atomic force microscopy (AFM) quantifies adhesion forces between *S. aureus*, lactobacilli, and epithelial cells, linking physical interactions to colonization and toxin production potential [111]. Purified TSST-1 and recombinant variants are used to assess endothelial cell responses, with ELISA quantifying cytokines (e.g., IL-6 and IL-8) and flow cytometry analyzing surface markers (VCAM-1 and ICAM-1) [112]. These methods provide mechanistic insights into toxin-induced host–pathogen interactions.

#### 3.3.5. MALDI-TOF MS

Matrix-assisted laser desorption/ionization time-of-flight mass spectrometry (MALDI-TOF MS) rapidly identifies microbial species by analyzing protein profiles [113,114,115]. However, it struggles to differentiate *Staphylococcus argenteus* from *S. aureus*, as both species share similar protein signatures. Whole-genome sequencing (WGS) overcomes this limitation, confirming the presence of the *tst-1* gene in *S. argenteus* isolates [114,116]. While MALDI-TOF MS remains a cornerstone for routine bacterial identification, its utility in distinguishing TSST-1-producing strains is limited without complementary genetic methods.

### 3.4. Combined Approaches and Validation and Quality Control

#### 3.4.1. Combined Molecular and Immunological Approaches

The detection of TSST-1 in *S. aureus* relies on integrated molecular and immunological techniques. PCR targeting the *tst* or *tst-1* gene is the cornerstone for genetic identification, offering high sensitivity and specificity [14,37,117]. Multiplex PCR assays enable the simultaneous detection of TSST-1 alongside other virulence genes (e.g., *sea* and *seb*), streamlining diagnostics in clinical and food samples [33,100]. For protein-level confirmation, ELISA and Western blotting are widely employed to quantify TSST-1 in culture supernatants or clinical specimens, with ELISA providing quantitative data (0.3–20 ng/mL detection range) and Western blotting offering qualitative validation [14,33,51]. Advanced approaches, such as luciferase-based transcriptional reporters and RNA sequencing (RNA-seq), further enhance kinetic and global transcriptional analyses [75]. WGS complements these methods by elucidating genomic contexts of TSST-1-positive isolates, revealing clonal associations (e.g., CC30) and virulence gene overrepresentation [95,118].

#### 3.4.2. Validation Strategies

The validation of TSST-1 detection assays emphasizes specificity, sensitivity, and reproducibility. Multiplex PCR protocols are rigorously validated using standard reference strains, with internal amplification controls (IACs) co-amplified to prevent false negatives [100]. Sequencing PCR products ensures genetic accuracy, as demonstrated in studies confirming *tst* amplicons against GenBank references [33,49]. Phenotypic validation includes correlating *tst* gene presence with functional toxin production; for example, TSST-1 levels quantified via ELISA in *tst*-positive isolates (0.3 ng/mL) confirm transcriptional activity [33,45]. Dual-tier validation, such as pairing promoter activity assays (luciferase reporters) with Western blotting, strengthens confidence in results [75]. Cross-method validation (e.g., combining PCR with PFGE or cytokine assays) addresses the limitations of single techniques, ensuring robust detection of genetic and functional toxin profiles [52,72].

#### 3.4.3. Quality Control Measures

Quality control (QC) in TSST-1 detection prioritizes minimizing false results and standardizing protocols (Table 3). Commercial kits, such as TST-RPLA (reverse passive latex agglutination), provide calibrated quantification (2.0 × 10^9^ CFU/mL detection threshold), ensuring reproducibility across laboratories [52]. For immunological assays, recombinant TSST-1 standards and anti-TSST-1 antibodies calibrate ELISA and Western blotting, establishing dynamic detection ranges (0.6–20 ng/mL) [119]. In molecular workflows, primer specificity checks and housekeeping gene normalization (e.g., *gyrB* in qRT-PCR) reduce technical variability [99,101]. Comparative genomic analyses against reference strains (e.g., ST72 clade D isolates) further ensure accurate virulence factor profiling [118]. Additionally, phenotypic–genotypic concordance testing, such as verifying TSST-1 protein absence in tst mutants via Western blotting, validates assay reliability [120]. These QC measures enhance diagnostic accuracy and are critical for managing TSS outbreaks and evaluating therapeutic interventions [121,122].

## 4. Structure of TSST-1

### 4.1. Molecular Characteristics of TSST-1

TSST-1 is a potent superantigen produced by *S. aureus*, a major TSS causative agent. TSST-1 is a 22–22.5 kDa single-chain protein consisting of approximately 234 amino acids [46,130]. Its primary structure is defined by the presence of a 40-residue signal peptide cleaved to yield the mature toxin, which is crucial for its immune-modulating activity [3]. This structure is highly conserved among staphylococcal superantigens, and the stability of TSST-1 under a wide range of environmental conditions is a critical feature contributing to its persistence in clinical, foodborne, and environmental settings [93,131].

TSST-1 is a compact, globular protein whose structural integrity is essential for its superantigenic activity. Crystallographic studies have elucidated a rigid three-dimensional structure, where the stability of TSST-1 is attributed to its specific folding patterns, including a centrally located β-sheet structure flanked by α-helices [132,133]. These structural elements contribute to TSST-1’s remarkable resistance to proteases, heat, and other harsh environmental conditions, which enhances its persistence in different ecological niches [46,134]. TSST-1’s stability also plays a significant role in its ability to remain functional in the human body, even in environments with low pH, such as the vagina during menstruation, contributing to its association with mTSS [117].

**Figure 1:**
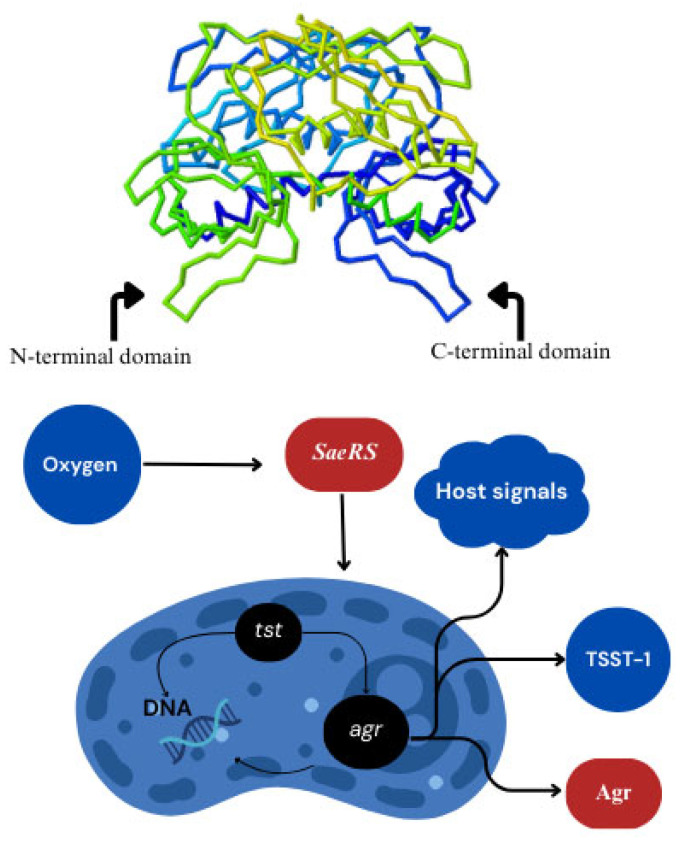
TSST-1 structure and regulation.

Moreover, the molecular stability of TSST-1 supports its prolonged persistence and survival in food matrices and clinical environments, which are key factors contributing to its spread (Figure 1). As TSST-1 remains stable in the environment, it increases the likelihood of contamination and transmission, making it a significant pathogen in clinical and foodborne settings [93,131].

### 4.2. Domain Architecture and Functionality

TSST-1’s domain architecture is pivotal in mediating its superantigenic properties, enabling it to bypass conventional antigen processing and trigger an excessive immune response. The structure of TSST-1 is divided into two main functional domains: the N-terminal domain and the C-terminal domain. Each domain plays a distinct role in the toxin’s interaction with the host immune system, a trait central to its pathogenic activity [135,136,137].

#### 4.2.1. N-Terminal Domain and MHC Class II Binding

The N-terminal domain of TSST-1 contains the oligonucleotide/oligosaccharide-binding (OB)-fold, a structural motif crucial for its interaction with MHC class II molecules on APCs. The OB-fold enables TSST-1 to engage directly with the β-chain of MHC class II, bypassing the usual antigen processing and presentation pathway [5]. This interaction is fundamental to TSST-1’s superantigenic activity, as it triggers an exaggerated immune response, leading to polyclonal T-cell activation. The ability of TSST-1 to interact with MHC class II molecules without needing antigen processing is a key feature that distinguishes it from conventional antigens [138,139].

The OB-fold also stabilizes the interaction between TSST-1 and APCs, ensuring that the toxin can bind with high affinity even in environmental stressors such as proteases, high temperatures, and acidic conditions. This stability is critical for TSST-1’s persistence in the human body and its ability to induce immune dysregulation over extended periods [131,134].

#### 4.2.2. C-Terminal Domain and TCR Binding

The C-terminal domain of TSST-1 binds to the TCR Vβ regions, specifically Vβ2, Vβ12-3, and Vβ12-4 [137]. This interaction is essential for the toxin’s ability to induce polyclonal T-cell activation. When TSST-1 binds to the TCR, it causes massive, non-specific activation of T cells, bypassing the normal antigen specificity typically observed in immune responses. This leads to the release of pro-inflammatory cytokines such as TNF-α, IL-6, and IFN-γ, which are responsible for the systemic inflammation seen in TSS [5,140]. The C-terminal domain contains a β-grasp motif, which enhances the specificity of the interaction between TSST-1 and TCRs, further increasing the efficiency of T-cell activation. Mutations in key residues, such as *Ser72*, can significantly impair this interaction, reducing TSST-1’s ability to trigger the cytokine storm that underpins TSS [1]. This underscores the importance of structural motifs in determining the potency of TSST-1’s superantigenic activity and its capacity to induce immune dysregulation [5].

The dual functionality of the N-terminal and C-terminal domains and the central β-barrel structure underpins TSST-1’s potent ability to induce the massive immune activation that characterizes TSS. These domains ensure the toxin’s immunological efficacy and provide insights into potential therapeutic strategies to interrupt immune dysregulation driven by TSST-1 [5].

#### 4.2.3. Central β-Sheet and β-Barrel Domain

In addition to the N-terminal and C-terminal domains, TSST-1 contains a central β-sheet or β-barrel domain, which serves as the structural core of the protein. This domain provides stability to TSST-1, ensuring that the protein can maintain its functional integrity under various conditions. The β-barrel structure allows the toxin to interact with MHC class II and TCRs efficiently, facilitating the formation of the ternary complex central to its superantigenic activity [138]. The flexibility of the loops in this domain ensures that the toxin can adapt its conformation to bind with high affinity to the immune receptors, further enhancing its ability to induce immune activation.

The structural integrity of the β-barrel domain is essential for TSST-1’s persistence in the host. It allows it to maintain its immunomodulatory function despite environmental stressors, such as temperature changes, protease exposure, or immune system responses. This resilience is a critical feature of TSST-1’s pathogenicity and is key in its ability to induce prolonged immune dysregulation [131].

### 4.3. Structural Comparisons with Other Superantigens

While TSST-1 shares structural features with other superantigens in the *S. aureus* family, such as SEB and SEA, several key differences set TSST-1 apart regarding its binding specificity and immunological effects.

Like other superantigens, TSST-1 contains a β-sheet-rich fold and β-barrel structures that are integral to its ability to bind both MHC class II molecules and TCRs. These structural elements are conserved across the superantigen family and enable the toxins to activate a wide range of T cells by binding to the TCRs regardless of antigen specificity [5,16]. However, TSST-1 exhibits several unique structural characteristics that contribute to its distinct functional properties.

One major difference between TSST-1 and other superantigens like SEB lies in SEB’s absence of the emetic cystine loop [137]. This loop is responsible for the gastrointestinal toxicity observed with SEB, including its ability to induce vomiting, a feature not shared by TSST-1. The absence of this loop explains why TSST-1 does not cause food poisoning symptoms such as emesis, despite sharing structural similarities with SEB [5]. This structural distinction highlights the differences in the biological activities of these toxins, despite their shared ability to trigger a systemic immune response.

Another key structural difference is the positioning of the TCR-binding site. In TSST-1, the TCR-binding site is located at the “top-back” of the molecule, while in SEB, the binding site is at the “top-front” [141]. This topographical difference in the positioning of the TCR-binding region influences receptor binding specificity. It may contribute to differences in T-cell subtypes preferentially activated by these toxins. The distinct receptor specificity patterns between TSST-1 and SEB result in variations in the cytokine profiles and immune responses that induce, which may explain the different clinical manifestations of the diseases they cause [142].

Moreover, the binding affinity of TSST-1 for MHC class II molecules appears to be higher than that of other superantigens. This increased affinity is attributed to the flexibility of the loop regions and the structural organization of TSST-1, which enhance its interaction with MHC class II compared to other superantigens like SEA and SEB [5,16]. These subtle differences in structure and binding affinity contribute to TSST-1’s exceptionally potent superantigenic activity, enabling it to induce a stronger immune response than other toxins in the superantigen family.

Furthermore, TSST-1’s interaction with TCRs is more restricted than that of other superantigens. While SEs can bind to a broad range of TCR Vβ regions, TSST-1 prefers binding to specific Vβ regions, including Vβ2. This specificity for certain TCR Vβ regions contributes to the severity of the immune response and TSS’s pathogenesis [143]. The selective interaction with Vβ2 TCRs may explain the particular cytokine profiles and immune responses observed with TSST-1, which are distinct from those triggered by other staphylococcal superantigens [15].

## 5. Mechanism of Action of TSST-1

The pathogenicity of *S. aureus* in the context of TSS is a complex and multi-step process driven by the production of TSST-1. The pathogenesis of TSST-1 can be encapsulated in a unifying model involving three interconnected stages: (1) superantigenic activity and immune dysregulation, (2) a cytokine storm and its pathophysiological consequences, and epithelial barrier disruption (3). Each of these stages plays a pivotal role in the rapid onset of severe clinical symptoms, including multi-organ failure and shock, and provides insights into how TSST-1 contributes to the devastating effects of TSS.

### 5.1. Superantigenic Activity and Immune Dysregulation

TSST-1 is an exceptionally potent superantigen produced by *S. aureus*. TSST-1 acts outside the normal antigen processing and presentation pathways as a superantigen. It binds directly to the major MHC-II molecules on antigen APCs. It simultaneously interacts with specific Vβ regions of TCRs. This direct binding, which bypasses the conventional antigen presentation process, activates a significant portion of the T-cell population, with up to 20% of T cells being activated irrespective of their antigen specificity [3]. This polyclonal activation contrasts with normal antigen-specific T-cell activation, leading to an uncontrolled and massive immune response. TSST-1 induces a cascade of cytokine release, including IL-2, TNF-α, and IFN-γ, which contribute significantly to the development of the “cytokine storm” [144,145].

The immune dysregulation that follows this cytokine storm leads to severe consequences for the host. The cytokine surge disrupts the normal homeostasis of the immune system, triggering a system-wide inflammatory response that affects multiple organs. The activation of many T cells without the regulatory mechanisms that usually control such activation results in excessive inflammatory mediator production. This dysregulated immune response can overwhelm the body’s ability to contain inflammation, leading to a systemic inflammation that causes extensive tissue damage [145]. This overwhelming activation of the immune system may also trigger the apoptosis of immune cells, further exacerbating the dysregulation [74]. This can lead to multi-organ failure in severe cases, as the immune system continues to target both infected and healthy tissues indiscriminately.

Moreover, the superantigenic nature of TSST-1 allows it to affect both Th cells and CTLs, resulting in an amplified immune response that leads to widespread cytokine release. The cross-linking between MHC-II and TCRs induces the release of more inflammatory cytokines, further amplifying the immune activation cascade. This persistent activation not only overwhelms the immune system but also perpetuates the inflammatory cycle, making it difficult for the body to regain control and leading to severe pathological consequences, such as septic shock and organ dysfunction [144].

### 5.2. Cytokine Storm and Pathophysiological Consequences

The cytokine storm triggered by TSST-1 is central to the pathophysiology of TSS. A hallmark of TSS is the massive, uncontrolled release of pro-inflammatory cytokines, such as TNF-α, IL-2, IL-6, IL-8, and IFN-γ, which leads to systemic inflammation [45,95,146]. This overproduction of cytokines causes widespread endothelial dysfunction, which leads to the leakage of fluid from blood vessels, a condition known as vascular leakage. This leakage, in turn, contributes to the hypovolemic shock characteristic of TSS, marked by a drop in blood pressure and a reduction in the perfusion of vital organs [70]. If not managed promptly, this shock can result in multi-organ failure, as reduced blood flow to the kidneys, liver, and lungs leads to their dysfunction.

The pathophysiological effects of the cytokine storm are extensive. The pro-inflammatory cytokines, particularly TNF-α, induce endothelial cell activation and increase the expression of adhesion molecules like ICAM-1 and VCAM-1. These molecules mediate the recruitment of additional immune cells to sites of infection, further exacerbating the inflammatory response. This continuous recruitment of immune cells amplifies cytokine release and creates a feedback loop that perpetuates systemic inflammation [74]. Moreover, the endothelial cells become increasingly permeable, allowing immune cells and other inflammatory mediators to penetrate tissues and organs, which leads to further tissue damage, including the destruction of vascular structures and organ failure [147].

The inflammatory cascade also causes significant tissue damage in various organs, including the heart, liver, lungs, and kidneys. Releasing cytokines like IL-6 and TNF-α leads to immune cell activation, which causes tissue destruction. For instance, the lung cytokine storm leads to pulmonary edema and respiratory distress syndrome (ARDS), where fluid accumulates in the alveoli, preventing proper gas exchange and causing hypoxia [148]. Similarly, in the kidneys, cytokine-induced inflammation can lead to renal failure, contributing to the systemic nature of TSS. These effects are compounded by the continuous activation of the immune system, leading to prolonged organ damage and dysfunction [149].

Endothelial damage also plays a key role in developing disseminated intravascular coagulation (DIC), which can result from excessive cytokine release. The endothelial cells lining blood vessels are activated by cytokines like TNF-α and IL-2, which promote platelet aggregation and coagulation. This leads to microthrombi formation throughout the vasculature, which can further impair blood flow to vital organs and contribute to the progression of organ failure [150].

### 5.3. Epithelial Interactions and Barrier Disruption

In addition to its immune-modulating effects, TSST-1 plays a significant role in disrupting epithelial barriers, contributing to TSS’s pathogenesis and amplifying its systemic spread. The primary cells affected by TSST-1 in this process are keratinocytes, the key epithelial cells of the skin and mucosal barriers. TSST-1 binds to CD40 receptors on keratinocytes, activating these cells and producing pro-inflammatory chemokines, such as IL-8, IL-33, and MIP-3α [16,151]. These chemokines recruit additional immune cells to the site of infection, further exacerbating the inflammatory response and enhancing tissue permeability.

One of the primary consequences of TSST-1’s interaction with epithelial cells is the disruption of tight junctions, which are essential for maintaining the integrity of epithelial barriers. By interfering with these tight junctions, TSST-1 increases epithelial permeability, making it easier for the toxin and other pathogens to invade deeper tissues and enter the bloodstream [151]. In the case of menstrual cup-associated TSS, for example, the disruption of the vaginal epithelium allows TSST-1 to enter the bloodstream, contributing to the systemic effects of the toxin [16]. Similarly, in conditions like eczema herpeticum, TSST-1-induced epithelial dysfunction enhances the skin’s susceptibility to secondary infections, including those caused by *Herpes simplex virus* (HSV), thereby exacerbating the inflammatory response [99].

Beyond facilitating the spread of TSST-1, epithelial disruption also creates a conducive environment for secondary bacterial infections. TSST-1 has been shown to alter the expression of adhesion molecules on epithelial cells, which can enhance the colonization and persistence of other microbial pathogens in compromised mucosal environments. This alteration in the expression of adhesion molecules, in conjunction with the increased permeability of epithelial barriers, creates a vicious cycle of inflammation, tissue damage, and susceptibility to further microbial invasion [151].

Thus, TSST-1’s effects on epithelial cells play a pivotal role in amplifying the severity of TSS, not only by promoting local inflammation but also by facilitating the toxin’s systemic dissemination [141]. The disruption of epithelial integrity is a key mechanism that enables TSST-1 to spread throughout the body, exacerbating its pathophysiological effects and increasing the risk of secondary infections [152,153].

### 5.4. Clinical Relevance and Host Factors

The clinical significance of TSST-1 extends beyond its role in the pathogenesis of TSS. It highlights the crucial influence of the host’s immune status and the specific virulence of the *S. aureus* strain producing the toxin [2,6]. TSST-1 is most frequently associated with severe infections caused by MRSA, particularly in immunocompromised individuals. Such individuals, including those with chronic kidney disease, diabetes, or those undergoing immunosuppressive therapy, are more susceptible to the toxic effects of TSST-1 due to their impaired immune responses. In these populations, the systemic effects of TSST-1 are often exacerbated, leading to more severe manifestations of TSS and an increased risk of mortality [154,155].

The severity of TSST-1-associated disease is also heavily influenced by the host’s immune response. Immunocompromised individuals, in particular, have a diminished ability to regulate the cytokine storm triggered by TSST-1, resulting in excessive and uncontrolled inflammation. This lack of immune regulation leads to a heightened risk of multi-organ failure, septic shock, and death [155,156]. Additionally, the presence of other virulence factors produced by *S. aureus*, such as PVL, can exacerbate the severity of the infection. PVL is known to cause tissue destruction and contribute to necrotizing pneumonia, further complicating the clinical course of TSST-1-induced TSS [157,158].

Another factor that influences the clinical outcome of TSST-1-associated disease is the type of infection and the presence of specific risk factors. Menstrual TSS is most commonly associated with the use of tampons, which provide an ideal environment for *S. aureus* to colonize and produce TSST-1 [159]. However, non-menstrual TSS can arise in individuals with surgical wounds, abscesses, or other localized infections [160,161]. In both forms of TSS, TSST-1 contributes to the rapid onset of systemic symptoms, including fever, hypotension, and multi-organ failure, highlighting the importance of early detection and intervention [73].

Moreover, the virulence of TSST-1 is not uniform across all *S. aureus* strains. Some strains, particularly those classified as hypervirulent, produce higher quantities of TSST-1, which leads to more severe disease presentations [162,163,164]. The genetic makeup of these strains, including mutations and the presence of other virulence factors, can significantly impact the clinical outcome. For instance, strains producing both TSST-1 and other toxins, such as α-toxin or β-hemolysin, often lead to more aggressive clinical manifestations, increasing the morbidity and mortality associated with the infection [5].

The host’s genetic makeup also plays a role in determining the severity of TSST-1-associated disease. Certain host genetic factors, such as variations in cytokine production or immune receptor expression, can influence the outcome of infection. For example, individuals with certain polymorphisms in cytokine genes may be more prone to an exaggerated inflammatory response, which increases the likelihood of developing severe symptoms [70].

## 6. Genetic Regulation of TSST-1

### 6.1. Regulation Systems

The production of TSST-1 in *S. aureus* is intricately regulated by a network of genetic and environmental factors. This regulation ensures that the pathogen can optimize toxin production based on environmental cues, bacterial population dynamics, and the host’s immune response [165]. The genetic systems that control TSST-1 production include the *agr* quorum-sensing system, the *SaeRS* two-component system, *sarA*, and regulatory proteins such as *Rot* and *SigB* [166,167]. These systems allow the bacterium to adapt and maximize its virulence in response to the host environment [152,168].

#### 6.1.1. *agr* System

The *agr* system is a crucial component of *S. aureus*’s regulatory network for virulence factor production, including TSST-1. *Agr* functions through a quorum-sensing mechanism, where the expression of target genes, including *tst* encoding TSST-1, is dependent on the bacterial population density [169,170,171,172]. As the population density increases, the *agr* system becomes activated, producing RNAIII, which acts as a key effector molecule in regulating virulence factors. RNAIII directly or indirectly modulates the transcription of the *tst* gene, ensuring that TSST-1 is produced appropriately, especially during the post-exponential growth phase when the bacterial population is high and the infection is well established [165,166].

Interestingly, even when the *agr* system is mutated, TSST-1 production may still occur, although it is often reduced. This indicates the compensatory role of other genetic regulators in maintaining virulence under conditions where *agr* is compromised [31]. This redundancy in regulatory control ensures that *S. aureus* can adapt to different growth phases and environmental challenges, even if one regulatory system is impaired [168].

#### 6.1.2. *SaeRS* Two-Component System

The *SaeRS* two-component system is another essential regulator of TSST-1 expression. *SaeR* is the response regulator, and *SaeS* is the sensor kinase [76,173]. This system is activated under certain stress conditions, such as low oxygen levels and high salt concentrations, which are typically found in certain host environments, like abscesses or mucosal surfaces [98]. *SaeR* binds directly to the *tst* promoter, promoting TSST-1 transcription. The activation of *SaeRS* under these conditions underscores the bacterium’s ability to respond to the hostile conditions found within host tissues [3,75].

#### 6.1.3. *sarA*, Rot, and SigB

The *sarA* regulator is a global regulator that influences a wide range of *S. aureus* virulence factors, including TSST-1. *SarA* has a dual role, repressing and enhancing TSST-1 production, depending on the strain and environmental context. It acts synergistically with *agr*, modulating TSST-1 production based on environmental stresses such as nutrient availability or oxygen levels [41,58].

The Rot protein functions as a repressor of TSST-1 production by binding to the *tst* promoter, preventing the excessive release of the toxin. This helps maintain a balance between bacterial survival and virulence factor production, ensuring that toxin production does not become detrimental to the bacterium under certain conditions [12].

*SigB* is an alternative sigma factor that is important in modulating the bacterial response to environmental stress [174,175]. *SigB*’s influence on TSST-1 production is largely indirect, as it modulates the activity of *agr* and *sarA*, contributing to the repression of TSST-1 expression in response to stress [137,176]. This regulation is critical in ensuring that the production of TSST-1 is tightly controlled, preventing it from being produced when the bacterium is under unfavorable conditions [3].

### 6.2. Environmental and Host-Dependent Modulation

The regulation of TSST-1 production is not solely controlled by genetic systems but is also strongly influenced by the external environment and host-specific factors [21,177]. The bacterium must continuously assess and adapt its gene expression in response to various physical and chemical cues in the environment and the host. These environmental factors include glucose and iron availability, oxygen tension, and pH, all of which significantly affect TSST-1 production [178].

#### 6.2.1. Glucose and Iron Availability

Glucose plays a crucial role in the regulation of TSST-1. Under conditions of high glucose availability, TSST-1 production is suppressed by CcpA (catabolite control protein A). This ensures that the bacterium does not waste energy producing virulence factors when abundant resources are available [179]. However, in conditions where glucose levels are low, such as during menstruation or when nutrients are scarce, this repression is relieved, and TSST-1 production is enhanced. This mechanism allows *S. aureus* to adapt its virulence factor production to the nutrient availability in its environment [11].

Iron availability is another critical factor influencing TSST-1 expression. In environments where iron is scarce, such as within the host’s immune system, *S. aureus* upregulates TSST-1 production [4]. This increased production helps the bacterium overcome the host’s iron sequestration mechanisms, allowing it to maintain its virulence. Conversely, when iron is plentiful, TSST-1 expression is repressed [127].

#### 6.2.2. Oxygen Tension and pH Levels

The bacterium’s response to oxygen availability is a key modulator of TSST-1 production. Low-oxygen environments, which are common in host niches like abscesses, provide conditions that activate the *SaeRS* two-component system and promote TSST-1 production [130]. Similarly, the pH of the surrounding environment influences TSST-1 expression. Acidic conditions, such as those found in inflamed tissues or the vaginal environment during menstruation, favor the production of TSST-1 [34,180]. These environmental factors work in concert with genetic regulatory systems to ensure that TSST-1 is produced at the right time and in the right location, maximizing the bacterium’s ability to cause disease [143,164,176].

#### 6.2.3. Mucosal Surfaces and Abscesses

Mucosal surfaces and abscesses are two key host niches where *S. aureus* can thrive and produce high levels of TSST-1. The combination of low oxygen, nutrient scarcity, and acidic pH conditions in these environments provides an ideal setting for TSST-1 production. In the case of vaginal mucosa, such as during menstruation, these conditions activate the *agr* and *SaeRS* systems, leading to increased TSST-1 expression [10,179]. Similarly, abscesses, which are low in oxygen and rich in cellular debris, provide an optimal environment for *S. aureus* to produce TSST-1 and other virulence factors, contributing to the severity of infection [58].

#### 6.2.4. Biofilms and Chronic Infections

Biofilm formation is another important aspect of *S. aureus* infection. Biofilms often form in devices like tampons, catheters, and prosthetic devices, where the bacteria can accumulate and produce high concentrations of TSST-1. These biofilms protect *S. aureus* from host immune responses and antimicrobial treatments, allowing the bacteria to persist in the host and exacerbating the disease’s severity [16,105].

In chronic infections, where *S. aureus* persists in biofilms or other host niches, TSST-1 levels can accumulate much higher than those observed in planktonic cultures. This accumulation can significantly contribute to the pathogenesis of diseases such as toxic shock syndrome, where high levels of TSST-1 in the bloodstream lead to systemic inflammation and immune system dysregulation [105].

### 6.3. Host Immune Interactions

Host immune signals also play a role in modulating TSST-1 production. The activation of immune receptors, such as toll-like receptors (TLRs), or the release of cytokines IL-6 in response to infection can influence the bacterial stress response and subsequently increase the production of TSST-1 [165,166]. This immune modulation adds a layer of complexity to the regulation of TSST-1, as it can enhance virulence through feedback loops involving both the bacterium and the host immune system [2,181].

### 6.4. Interactions with Resistance and Co-Regulation

The expression of TSST-1 in *S. aureus* is often linked to the bacterium’s antibiotic resistance profile. This correlation suggests that regulating virulence factors such as TSST-1 may be intertwined with mechanisms that help the bacterium survive in the presence of antibiotics [165]. One of the key resistance mechanisms in *S. aureus* is acquiring the *mecA* gene, which confers resistance to methicillin. MRSA strains are frequently observed to carry the *tst* gene, but interestingly, not all of these strains produce TSST-1 in significant amounts. This observation points to a potential trade-off between the production of virulence factors and the energy demands of antibiotic resistance mechanisms [182].

The co-regulation of virulence and resistance genes may be an adaptive strategy that allows *S. aureus* to maximize its survival and pathogenicity. For instance, when exposed to antibiotic pressure, *S. aureus* may prioritize the expression of resistance genes like *mecA* over virulence factors such as TSST-1 as a way to conserve energy and resources for survival under hostile conditions [183]. This is seen in the case of MRSA strains, where TSST-1 production is often suppressed under certain conditions of antibiotic stress [184]. However, when these same strains encounter a host environment, the regulation of TSST-1 may be re-activated, particularly if the bacterium faces conditions that favor toxin production, such as low oxygen or nutrient scarcity [178].

The complex relationship between TSST-1 and antibiotic resistance further complicates treatment strategies. In MRSA infections, the presence of TSST-1 not only exacerbates the disease’s severity but also complicates efforts to control the infection due to the bacteria’s ability to resist common antibiotics [185,186]. The coexistence of both virulence and resistance traits highlights the dual threat posed by MRSA, as these strains are often more difficult to treat due to their resistance to multiple antibiotics and their enhanced ability to cause severe disease [39,184,187,188].

Furthermore, the interaction between TSST-1 and other resistance mechanisms, such as β-lactamase production (via the *blaZ* gene), suggests a coordinated regulatory network that not only facilitates bacterial survival in the presence of antibiotics but also enhances the strain’s pathogenicity [183,189]. These resistance mechanisms and virulence factors are often co-regulated, providing *S. aureus* with a competitive advantage in hospital and community-acquired infections [30,102].

### 6.5. Strain-Specific Variations and Horizontal Gene Transfer

The regulation of TSST-1 production is not uniform across all *S. aureus* strains. There are significant strain-specific variations in the expression of the *tst* gene, which result in differing levels of TSST-1 production among isolates [190]. This variability can be influenced by genetic factors unique to each strain, including the presence of specific regulatory elements, genetic mutations, or environmental factors that affect gene expression. For example, in isolates from mastitis-infected cattle, TSST-1 production was detected in only one of several *S. aureus* strains, indicating that the ability to produce TSST-1 may be restricted to certain lineages or clonal types [33].

These strain-specific differences are not limited to TSST-1 expression alone. *S. aureus* is genetically diverse, with different clonal complexes (CCs) exhibiting varying virulence factor profiles, including differences in the production of other toxins, adhesins, and surface proteins [191,192]. Some clonal lineages, such as those associated with hospital-acquired infections, may be more virulent and capable of producing high levels of TSST-1. In contrast, others may make little to no TSST-1, depending on their genetic makeup [193].

Moreover, horizontal gene transfer (HGT) plays a crucial role in spreading virulence factors like TSST-1 between strains of *S. aureus*. Mobile genetic elements, such as plasmids and pathogenicity islands (*SaPI*s), are key mediators of this transfer. *SaPI*s, in particular, are genetic elements that carry virulence genes, including *tst-1*, and can be horizontally transferred between *S. aureus* strains through bacteriophage-mediated mechanisms [194]. This transfer facilitates the rapid spread of TSST-1-producing capabilities across different strains and species of *Staphylococcus*, enhancing the pathogenic potential of these bacteria [195].

The dissemination of TSST-1 production via HGT contributes to the epidemiological spread of this virulence factor. For example, MRSA strains carrying tst-1 can acquire or transfer the gene to other strains, leading to the emergence of new, more virulent clones. This is particularly concerning in hospital outbreaks, where the movement of bacterial strains between patients and medical devices can lead to the rapid dissemination of virulence factors such as TSST-1 [92].

Additionally, *S. aureus* strains that carry mobile resistance elements such as [36], which confer methicillin resistance, often also harbor tst-1, further complicating infection control measures. This genetic integration suggests that resistance and virulence factors can co-evolve, with horizontal gene transfer playing a significant role in shaping the genetic landscape of *S. aureus* and its pathogenicity [195].

## 7. Dissemination, Pathogenesis, and Clinical Impact of TSST-1

The dissemination of TSST-1-producing *S. aureus* strains is extensive, encompassing clinical, animal, and environmental reservoirs, thereby complicating control measures and highlighting the need for robust public health surveillance [196]. Clinically, TSST-1 is associated with severe infections caused by MRSA and MSSA strains. Infections linked to TSST-1-producing *S. aureus* include TSS, wound infections, sepsis, and neonatal bacteremia [152,162]. While MRSA strains are predominantly implicated in hospital-acquired infections (HAIs), TSST-1-producing MSSA strains are frequently involved in community-associated infections (CAIs), particularly among individuals with predisposing factors such as trauma, surgical interventions, or menstruation [64,148]. Although MRSA remains the principal TSST-1 producer in healthcare settings, the contribution of MSSA strains in community settings is significant, especially in skin and soft tissue infections leading to TSS [64]. Certain MRSA clones, notably ST22, demonstrate heightened virulence and are associated with severe clinical outcomes, including necrotizing pneumonia and septic shock, due to the combined effects of TSST-1 and other virulence determinants such as PVL [71]. Beyond human clinical infections, TSST-1-producing strains present considerable zoonotic risk, with frequent isolation from livestock and food products, particularly dairy and meat. This environmental presence emphasizes the potential for foodborne and occupational transmission, further expanding the public health impact of these strains [95].

### 7.1. Environmental Sources and Foodborne Transmission

TSST-1 has been detected in various environmental sources, including mastitis milk from dairy cattle, suggesting that livestock can serve as a reservoir for this toxin [96]. This raises significant concerns, as dairy workers and individuals involved in food processing may be exposed to TSST-1 through direct contact with contaminated animal products, posing a risk of zoonotic transmission. Furthermore, TSST-1 has been implicated in foodborne outbreaks, particularly where food products are improperly handled or stored [197,198]. Its stability across a wide range of temperatures enhances its persistence in food, making it a critical concern for food safety [95]. In clinical environments, TSST-1-producing *S. aureus* strains are frequently associated with severe infections such as neonatal bacteremia and toxic shock syndrome. These strains are particularly prevalent in intensive care units (ICUs), where immunocompromised patients are at heightened risk [23,118]. The ability of TSST-1-producing strains to form biofilms on medical devices, such as catheters and prosthetics, further complicates eradication efforts, enhancing their persistence and resistance to antimicrobial treatments [185]. Hospital-associated outbreaks involving TSST-1-producing strains have affected patients and healthcare workers, underscoring the critical need for stringent infection control practices, especially in ICU settings [199]. Importantly, the zoonotic and environmental reservoirs of TSST-1-producing *S. aureus* strains are not geographically confined; they have been reported globally, including in Asian countries such as India and China, where specific lineages like ST22 are associated with high virulence and multidrug resistance [64,70]. The emergence and spread of these resistant strains complicate infection management in both healthcare and community contexts. Given the substantial zoonotic potential of TSST-1-producing strains, it is essential to implement control strategies across clinical and agricultural sectors. Improving hygiene practices in livestock farming, particularly in dairy production, and ensuring proper food storage and handling are crucial measures to mitigate the risks of zoonotic and foodborne transmission [96].

### 7.2. Infection Control and Surveillance Measures

In healthcare settings, maintaining strict infection control protocols, including sterilizing medical devices and proper wound care, is critical for preventing the transmission of TSST-1-producing *S. aureus* strains [200]. Antimicrobial agents, such as linezolid and clindamycin, and infection prevention strategies can help reduce the incidence of hospital-acquired infections caused by these resistant strains [23]. Furthermore, global surveillance programs to monitor the spread of TSST-1-producing strains, particularly those exhibiting multidrug resistance, are essential for early detection and containment [95,96]. Tracking their prevalence in clinical and foodborne environments enables public health authorities to implement targeted interventions to mitigate associated risks.

### 7.3. Animal and Environmental Reservoirs

TSST-1-producing *S. aureus* strains are not restricted to human isolates. Still, they are also prevalent in various animal reservoirs, including cattle, pigs, and chickens, where they are often isolated from animals with chronic infections such as mastitis [201,202]. In dairy cattle, *S. aureus* is a well-established cause of bovine mastitis. While enterotoxins are more commonly detected, certain strains also produce TSST-1, highlighting the zoonotic potential of these pathogens. Transmission to humans can occur through direct contact with infected animals or through the consumption of contaminated animal products, particularly milk and dairy products [203,204].

Additionally, TSST-1-producing strains have been detected in poultry, raising concerns about foodborne transmission pathways. The presence of these strains in livestock further emphasizes the role of animals as reservoirs for *S. aureus*, with agricultural settings representing significant points of zoonotic transmission [205,206].

Beyond animal reservoirs, TSST-1-producing *S. aureus* strains have been isolated from various environmental sources, including wastewater systems, public spaces, and inanimate objects such as banknotes and mobile phones [207]. Environmental contamination with *S. aureus*, especially in regions with poor sanitation, presents a considerable risk for pathogen dissemination. Studies demonstrate that *S. aureus* can persist in wastewater systems, serving as a long-term reservoir for TSST-1-producing strains [205].

The ability of these strains to survive on surfaces such as hospital equipment and public fixtures complicates infection control efforts, creating opportunities for indirect transmission. Consequently, maintaining rigorous hygiene and disinfection practices in both public spaces and healthcare settings is essential to curtail the spread of *S. aureus* and its associated toxins [208,209,210].

### 7.4. TSST-1 and Its Role in TSS

#### 7.4.1. TSST-1 in Consumer Products and Public Health Implications

TSST-1 is closely associated with consumer products, particularly tampons, which represent the most commonly identified source of menstrual TSS. *S. aureus* strains producing TSST-1 can colonize tampons and other menstrual products, creating an optimal environment for bacterial proliferation and toxin production [159]. While tampon use remains the primary risk factor for menstrual TSS, other hygiene products, such as menstrual cups, have also been implicated as reservoirs for TSST-1-producing strains [83,151]. The widespread use of these products increases the likelihood of sporadic exposure, leading to severe infections in susceptible individuals.

Beyond consumer products, TSST-1-producing *S. aureus* strains have been detected in contaminated food products, including dairy items such as goat cheese [24,148]. Foodborne transmission highlights the importance of maintaining strict hygiene during food production and processing to prevent the ingestion of TSST-1 and subsequent infection [211,212].

The widespread distribution of TSST-1-producing strains across clinical, animal, environmental, and consumer product reservoirs underscores the urgent need for comprehensive public health surveillance. Routine monitoring of food products, clinical samples, and environmental sources is essential for the early detection and control of these strains. Surveillance efforts should be intensified in high-risk environments, including hospitals, agricultural facilities, and public spaces, to mitigate the risk of outbreaks [64].

Moreover, raising awareness about the risks associated with TSST-1 exposure among high-risk groups such as healthcare workers, livestock handlers, and menstruating individuals is vital for effective prevention. Public health interventions should extend beyond clinical management to address environmental and foodborne transmission pathways [213]. Emphasizing strict hygiene practices, regularly disinfecting hospital equipment, and adherence to food safety protocols are crucial strategies for reducing the spread of TSST-1-producing *S. aureus*. By implementing comprehensive prevention measures targeting multiple reservoirs, public health systems can significantly reduce the incidence of TSS and its associated morbidity and mortality [214].

#### 7.4.2. Menstrual vs. Non-Menstrual TSS

TSST-1 plays a crucial role in the pathogenesis of TSS and other severe infections. TSST-1 is a key virulence factor in both menstrual and non-menstrual forms of TSS, inducing systemic immune dysregulation that results in life-threatening symptoms such as fever, hypotension, and multi-organ failure. The production of TSST-1 in *S. aureus* is heavily influenced by host factors, environmental conditions, and bacterial virulence mechanisms [3,4,28,48].

TSS can manifest in both menstrual and non-menstrual forms, with distinct etiologies, though both are driven by the production of TSST-1 by *S. aureus*.

In menstrual TSS, *S. aureus* colonizes the vaginal mucosa, which is often facilitated by tampon use, though menstrual cups have also been implicated in the production of TSST-1 [24,215]. The conditions created by tampon or menstrual cup use, such as a low glucose, high oxygen, and near-neutral pH environment, favor the expression of the *tst* gene, which encodes TSST-1. The toxin is produced in the vaginal environment and breaches the mucosal barrier. It binds to epithelial cells, specifically CD40, which triggers the release of chemokines that recruit immune cells to the site. This leads to a systemic immune response as TSST-1 enters the bloodstream. The toxin further promotes the activation of immune cells and cytokine release, contributing to the development a cytokine storm [107].

Once TSST-1 reaches the bloodstream, it induces widespread immune activation and systemic inflammation, resulting in the characteristic signs of menstrual TSS: fever, rash, hypotension, and shock. This progression is directly related to the production and release of inflammatory mediators, which leads to severe systemic consequences.

In non-menstrual TSS, TSST-1 is similarly produced by *S. aureus* but in the context of invasive infections rather than tampon use. These infections often occur in tissues such as surgical wounds, burns, or abscesses, where *S. aureus* colonizes and produces TSST-1. While the toxin similarly enters systemic circulation, the clinical presentation in non-menstrual TSS may differ due to the diversity of potential infection sites. The onset of symptoms may be delayed, and diagnosis can be more challenging due to the lack of a clear trigger like tampon use [38,72,112]. Non-menstrual TSS may also be less readily diagnosed, as the clinical signs overlap with other systemic infections, making it difficult to identify and treat the condition promptly.

## 8. Treatment and Prevention of TSST-1

### 8.1. Antibiotic Therapy

The management of TSST-1-related infections requires antibiotics that inhibit *S. aureus* growth and suppress the production of TSST-1. Clindamycin and linezolid are central to this approach. Clindamycin is particularly effective due to its dual action: inhibiting bacterial protein synthesis and reducing TSST-1 production by interfering with the transcription of the *tst* gene [3,4]. It is highly effective, especially in regions with low antibiotic resistance rates, such as Morocco and Ethiopia [39]. However, resistance to clindamycin is emerging, particularly in the Middle East, highlighting the need for ongoing susceptibility testing [35].

Linezolid, another key antibiotic, is preferred for MRSA infections. Its ability to suppress TSST-1 production while controlling bacterial growth makes it invaluable in severe cases [216]. However, resistance to linezolid, particularly in strains resistant to other antibiotics, has been reported, necessitating careful monitoring. Vancomycin, though a first-line agent for MRSA, has reduced efficacy due to the rise in vancomycin-intermediate *S. aureus* (VISA) strains, and alternative treatments like daptomycin are used in such cases [217].

The emergence of multidrug-resistant strains of *S. aureus* complicates the management of TSST-1-producing infections, necessitating tailored antibiotic regimens based on local resistance patterns [35]. Regional antibiotic stewardship programs and continuous surveillance are essential to optimize treatment choices and control the spread of resistant strains.

### 8.2. Immunotherapy and Adjunctive Therapies

Immunotherapy is an essential adjunct to antibiotic treatment, particularly in severe cases of TSS. Intravenous immunoglobulin (IVIG) is widely used due to its ability to neutralize TSST-1 and reduce systemic inflammation by preventing the cytokine storm that is characteristic of severe TSS [2,107]. However, its high cost and the lack of clear guidelines regarding dosage and timing limit its use, and its efficacy in improving survival rates remains controversial [107].

Monoclonal antibodies (mAbs) targeting TSST-1 have demonstrated promising results in preclinical models. These antibodies block the interaction between TSST-1 and MHC class II molecules on antigen-presenting cells, thereby preventing T-cell activation and the release of inflammatory cytokines that drive TSS pathogenesis [1,23]. The preclinical success of mAbs such as MS473 paves the way for their inclusion in clinical trials.

Additionally, cytokine inhibitors, including anti-TNF-α and anti-IL-6 monoclonal antibodies, are under investigation for their ability to mitigate the hyperinflammatory response induced by TSST-1. Early studies suggest that these agents can reduce the severity of TSS by controlling cytokine production and mitigating organ damage [104]. Complementary therapies, such as corticosteroids and vitamin C, have also been explored for their potential to regulate the immune response and reduce oxidative stress in TSS patients [2].

### 8.3. Antivirulence Strategies

Antivirulence strategies offer a novel approach to managing TSST-1-producing *S. aureus* infections by targeting the virulence factors that mediate host damage. Rather than killing the bacteria, these strategies aim to inhibit the production of TSST-1 and other harmful toxins, thereby reducing the severity of infection and mitigating the need for traditional antibiotics [28,198,199,200,201,202,203,204,205,206,207,208,209,210,211,212,213,214,215,216,217,218,219].

Inhibiting TSST-1 production represents a promising antivirulence approach. *SaeRS* kinase inhibitors, such as phenazopyridine hydrochloride (PP-HCl), can suppress the expression of the *tst* gene and reduce TSST-1 production without affecting bacterial growth. This strategy is particularly beneficial in preventing menstrual TSS, as it allows for the reduction in toxin production while maintaining a healthy vaginal microbiota [27,75].

Monoclonal antibodies against TSST-1 offer another antivirulence strategy by preventing the interaction between the toxin and host immune cells, which is critical for the cytokine storm characteristic of TSS [11,23]. Additionally, small-molecule inhibitors and probiotics are being explored to interfere with the host–pathogen interaction and restore microbial balance, offering further options for mitigating TSST-1-induced damage [151,179].

### 8.4. Vaccine Development

Vaccine development against TSST-1 aims to prevent TSS and other toxin-mediated diseases caused by *S. aureus*. Toxoid-based vaccines, which use inactivated TSST-1 to stimulate an immune response, are a common approach. These vaccines have shown potential in animal models, eliciting protective antibodies that neutralize TSST-1 [3,9]. Another promising approach involves multi-component vaccines targeting multiple staphylococcal toxins, including TSST-1, PVL, and alpha-toxin, to provide broader protection against *S. aureus* infections [220,221].

Despite these promising developments, challenges remain in vaccine development, particularly due to the diversity of *S. aureus* strains and their ability to evade the immune system [20]. The complexity of *S. aureus* virulence factors requires comprehensive vaccines capable of generating strong and broad immunity to reduce the burden of TSST-1-mediated diseases [157].

### 8.5. Prevention Strategies

The prevention of TSST-1-related infections, especially TSS, involves a combination of hygiene practices, decolonization strategies, and vaccination. Hygiene practices, particularly during menstruation, are crucial for reducing the risk of TSST-1 exposure. Regularly changing menstrual products and using alternatives such as menstrual cups can help prevent TSS [24].

Decolonization protocols, including mupirocin for nasal decolonization and chlorhexidine washes for skin decolonization, are essential for reducing *S. aureus* colonization, particularly in high-risk individuals such as healthcare workers and patients undergoing surgery [222]. Probiotics, such as *Lactobacillus* species, are being investigated for their role in maintaining microbiota balance and preventing *S. aureus* overgrowth [179].

Additionally, antimicrobial stewardship programs are essential to control the spread of resistant *S. aureus* strains and minimize the risk of severe infections. The surveillance of TSST-1-producing strains and infection rates in healthcare settings ensures the timely implementation of control measures [104].

## 9. Futures Directions

Future research on TSST-1-producing *S. aureus* should focus on several key areas to improve diagnostic methods, therapeutic strategies, and our understanding of its pathogenesis and epidemiology [223].

First, improving diagnostic methods for TSST-1-producing strains is crucial. There is a need for more sensitive and specific diagnostic tools, such as advanced PCR assays, mass spectrometry, and biosensors. These tools would enable the rapid detection of TSST-1 in clinical and environmental samples, aiding early diagnosis and intervention. Additionally, integrating these diagnostic techniques into routine clinical practice, especially for high-risk populations, will significantly enhance detection and treatment efforts [9,224].

Vaccine development targeting TSST-1 remains a top priority. Efforts should be directed at optimizing toxoid vaccines and assessing their long-term efficacy in preventing superantigen-mediated diseases like TSS. Additionally, research should explore the broader impact of TSST-1 vaccines, particularly in preventing other *S. aureus* infections that this toxin could influence. Understanding the molecular mechanisms of TSST-1 and its interaction with the host immune system will be essential for developing effective vaccines [46,224].

Therapeutic strategies for TSST-1-related infections should focus on immunotherapeutic approaches, such as IVIG and monoclonal antibodies specifically targeting TSST-1. Moreover, developing small-molecule inhibitors to block TSST-1’s interaction with immune cells could provide an innovative therapeutic avenue. Understanding how TSST-1 production is regulated, especially through the *agr* system and other regulatory pathways, will help identify novel therapeutic targets. These therapies could mitigate the severe immune dysregulation associated with TSST-1 [224,225].

Future research should also investigate the molecular mechanisms regulating TSST-1 production. Understanding how environmental factors, such as pH, temperature, and host immune responses, influence TSST-1 expression is important. Additionally, investigating how TSST-1 interacts with host immune receptors, including CD40 and IL-10, could provide new insights into immune dysregulation and lead to targeted therapies to reduce the toxin’s harmful effects [215].

Another critical research priority is expanding the molecular surveillance of TSST-1-producing strains in clinical and community settings. This surveillance should include healthcare environments, foodborne outbreaks, and animal populations. Studies should focus on identifying the genetic and environmental factors that influence TSST-1 expression in different regions, as well as understanding the dynamics of TSST-1 transmission between humans and animals [226]. The surveillance of livestock-associated MRSA (LA-MRSA) strains, in particular, will be important in controlling the spread of TSST-1-producing strains in both human and animal populations [140,216].

Finally, future research should focus on the genetic diversity of TSST-1-producing *S. aureus* strains and the factors regulating the expression of the *tst* gene [164,176]. Investigating the role of mobile genetic elements, such as pathogenicity islands, in disseminating TSST-1 will provide insights into how this toxin spreads across bacterial populations [124,227]. Furthermore, studying the regulatory networks that control TSST-1 production, including environmental triggers and host-specific immune responses, will be crucial for identifying novel therapeutic strategies to prevent or mitigate the effects of this superantigen [75,221].

## 10. Conclusions

TSST-1 remains a critical virulence factor in *S. aureus*, which is primarily responsible for TSS through its superantigenic activity. Binding to MHC class II and T-cell receptors triggers a cytokine storm, contributing to severe systemic effects. TSST-1’s regulation is influenced by genetic and environmental factors, making its expression complex and context-dependent.

The emergence of TSST-1-producing MRSA strains poses significant public health challenges, with increased mortality rates and resistance to treatment. While antibiotics are vital, new therapeutic strategies, including monoclonal antibodies and vaccines targeting TSST-1, show promise.

Ongoing research into TSST-1’s structure, regulation, and immune interactions is essential for advancing diagnostics, treatments, and prevention strategies. Addressing the global impact of TSST-1-producing strains is crucial, especially given the rise in multidrug-resistant *S. aureus*.

## Figures and Tables

**Table 1:** Global prevalence of TSST-1-producing *S. aureus* strains across diverse populations and sample types.

Country	TSST-1 Prevalence (%)	Population Studied	Reference
Afghanistan	68.4	Non-medical university students (nasal)	[7]
Algeria	18.5	Clinical/environmental samples	[27]
Algeria	19.8	Isolates from humans, farm animals, pets, wildlife, and the environment	[28]
Benin	7.14	Hospital environment isolates	[29]
Brazil	0	Artisanal coalho cheese	[30]
Brazil	2.6	Raw milk, cheese, and cheese handlers in artisanal production	[31]
Brazil	52.08	Dairy products (Minas Frescal cheese)	[32]
Central Russia	1.6	Cows with subclinical mastitis	[33]
China	3.2	Bovine mastitis cases	[34]
China	3.8	CA-MRSA clinical infections	[35]
China	18	MRSA isolates (CC5 clone) from hospital patients in Suzhou	[36]
Egypt	18	Food samples (beef luncheon and corn flakes)	[37]
Egypt	21	Bacteremic and infective endocarditis patients	[38]
Ethiopia	13.37	Clinical specimens (wound, blood, etc.)	[39]
Ethiopia	51	Dairy products and milk samples	[40]
Hong Kong	9.52	Swine (pig tongues)	[41]
Iran	15.2	Patients with skin lesions	[20]
Iran	20.6	MS patients (nasal)	[42]
Italy	5.9	*S. aureus* from small ruminants (milk)	[43]
Japan	0.4	Healthcare facility patients	[44]
Japan	33.6 (2010), 21.6 (2018)	MRSA isolates from outpatient skin/pus samples	[45]
Kenya	23.3	Inpatients in referral hospital	[46]
Myanmar	3.5	Healthy food handlers	[4]
Nigeria	44.7	Livestock samples	[47]
Nigeria	3.3 (Nasal); 6.7(Clinical)	Nasal carriers and clinical patients (blood, wound, and sputum)	[48]
Poland	5	Free-living carnivorous mammals	[49]
Poland	3.5	Poultry (broiler chickens and turkeys)	[50]
Poland	8	Atopic dermatitis patients	[51]
Portugal	4.8	Raw milk isolates	[9]
Russia	58	HA-MRSA isolates (healthcare settings)	[52]
South Korea	13.3	Bovine mastitis milk samples	[53]
Uganda	0	Women in labor	[54]
United Kingdom	41	nmTSS cases (skin/soft tissue infections)	[55]
USA	4	Tampons colonized by *S. aureus*	[56]
USA	5	Vaginal colonization (tampon users)	[1]

**Table 2:** Comparative analysis of morbidity and mortality in TSS.

Region	Population	Morbidity	Mortality	Source
Quebec	630 TSS patients vs. 11,309 controls	Higher rehospitalization (men: 642.8 vs. 237.1 per 10,000); renal, hepatic, cardiovascular, neurological, and autoimmune risks increased.	Not directly reported	[81]
UK	88 children with TSS	Streptococcal TSS: 40% cardiovascular dysfunction and 25% renal failure	Overall: 5.7%; Streptococcal: 14.3%; Staphylococcal: 2.6%	[72]
USA	Patients <21 y, insured via Medicaid or commercial plans	ICU admission: 55–66%	Medicaid: 0%; Commercial: 0.5%	[88]
The Netherlands	Nationwide US adult and child cohorts	30.8% chronic outcomes: renal (10.2%), cardiovascular (8.3%), and autoimmune (3.9%)	2% to 38% depending on the case type	[84]
	67-year-old male case (necrotizing fasciitis)	Multi-organ failure and rapid deterioration	Fatal outcome	[89]
USA	US surveillance 1979–1996	Not specified	Menstrual: 5–15%; Non-menstrual: 30–50%	[86]
Japan	National cohort of STSS cases	28% acute renal failure	STSS responsible for >60% of TSS deaths	[90]

**Table 3:** Comparative assessment of methods for detecting the TSST-1 toxin and the *tst* gene in *S. aureus*.

Method	Type	Principle	Accuracy	Advantages	Inconveniences	References
PCR (tst gene)	Molecular	Amplifies the tst gene using specific primers.	Sensitivity: >90%. Specificity: High. Detection limit: ~10^2^ CFU/g.	Rapid, specific, and high throughput.	Requires DNA extraction; risk of false negatives if primers mismatch.	[20,27,91,92,102,123,124]
Multiplex PCR	Molecular	Simultaneously amplifies tst with other toxin genes (e.g., sea and seb).	Sensitivity: 95–100%. Specificity: High.	Efficient for multi-toxin screening.	Complex primer design; risk of cross-reactivity.	[50,96]
Real-time PCR	Molecular	Quantifies tst mRNA using fluorescent probes.	Sensitivity: ~1–10 copies/µL.	Quantitative, rapid, and minimal post-processing.	Expensive equipment; requires probe optimization.	[31,55,93,125]
qRT-PCR	Molecular	Quantifies tst mRNA expression using reverse transcription.	Sensitivity: ~10 mRNA copies.	Links gene expression to toxin production	Requires RNA extraction; risk of RNA degradation	[31,119,121]
ELISA	Immunological	Uses anti-TSST-1 antibodies to detect the toxin in samples.	Sensitivity: 0.6–20 ng/mL. Specificity: High.	High-throughput, quantitative, and user-friendly.	Cross-reactivity with related toxins; requires purified standards.	[24,87,98,126]
Western blotting	Immunological	Detects the TSST-1 protein using specific antibodies and SDS-PAGE.	Sensitivity: 1–10 ng. Specificity: Very high.	Confirms protein identity; semi-quantitative.	Time-consuming; requires skilled personnel.	[28,51,74,112,127,128]
Mass Spectrometry	Proteomic	Identifies TSST-1 via peptide mass fingerprinting.	Sensitivity: <1 ng/mL. Specificity: Very high.	Detects post-translational modifications; no antibodies needed.	Expensive; requires advanced equipment and expertise.	[3,21,41]
Bioassay (T-cell activation)	Functional	Measures IL-2 release from PBMCs exposed to TSST-1.	Sensitivity: ~0.02 ng/mL (functional activity).	Confirms biological activity; useful for toxin validation.	Time-intensive; requires cell cultures; low throughput.	[72]
Latex Agglutination	Immunological	Detects TSST-1 via antibody-coated latex particles.	Sensitivity: 1–2 ng/mL. Specificity: Moderate.	Rapid (15–20 min); no specialized equipment.	Lower sensitivity; prone to false positives in mixed samples.	[52,97,104,105]
Whole-Genome Sequencing	Molecular	Identifies tst gene in bacterial genomes.	Sensitivity: 100% (gene presence). Specificity: High.	Comprehensive; detects mutations and genetic context.	Expensive; computationally intensive; not routine for diagnostics.	[45,53,64,71,129]

## Data Availability

No new data were created or analyzed in this study.

## Abbreviations

TSST-1	Toxic Shock Syndrome Toxin-1
TSS	Toxic Shock Syndrome
MRSA	Methicillin-Resistant *Staphylococcus aureus*
MSSA	Methicillin-Sensitive *Staphylococcus aureus*
MHC-II	Major Histocompatibility Complex Class II
TCR	T-cell Receptor
PCR	Polymerase Chain Reaction
ELISA	Enzyme-Linked Immunosorbent Assay
WGS	Whole-Genome Sequencing
qPCR	Quantitative Real-Time PCR
MALDI-TOF MS	Matrix-Assisted Laser Desorption/Ionization Time-of-Flight Mass Spectrometry
IVIG	Intravenous Immunoglobulin
PVL	Panton–Valentine Leukocidin
DIC	Disseminated Intravascular Coagulation
ARDS	Acute Respiratory Distress Syndrome
IL	Interleukin
TNF-α	Tumor Necrosis Factor-alpha
IFN-γ	Interferon-gamma
ST	Sequence Type
CC	Clonal Complex
VISA	Vancomycin-Intermediate *Staphylococcus aureus*
HGT	Horizontal Gene Transfer
LA-MRSA	Livestock-Associated Methicillin-Resistant *Staphylococcus aureus*
CD40	Cluster of Differentiation 40
ICAM-1	Intercellular Adhesion Molecule 1
VCAM-1	Vascular Cell Adhesion Molecule 1
Agr	Accessory Gene Regulator
SaeRS	Two-component regulatory system
Rot	Repressor of Toxins
SigB	Sigma Factor B
CcpA	Catabolite Control Protein A
RPLA	Reversed Passive Latex Agglutination
LC-SRM	Liquid Chromatography-Selected Reaction Monitoring
AFM	Atomic Force Microscopy
SDS-PAGE	Sodium Dodecyl Sulfate-Polyacrylamide Gel Electrophoresis
SAgs	Superantigens
AD	Atopic Dermatitis
ICU	Intensive Care Unit
CA-MRSA	Community-Associated Methicillin-Resistant *Staphylococcus aureus*
HAIs	Hospital-Acquired Infections
LA-MRSA	Livestock-Associated Methicillin-Resistant *Staphylococcus aureus*
